# Harnessing GSK-3β inhibition for lung cancer therapy: emerging opportunities and challenges

**DOI:** 10.1007/s12032-025-03086-5

**Published:** 2025-11-11

**Authors:** Emad H. M. Hassanein, Hanan S. Althagafy, Hanan H. Abd- ElHafeez, Islam M. Ibrahim, Badrah S. Alghamdi

**Affiliations:** 1 Department of Pharmacology and Toxicology, Faculty of Pharmacy, Al-Azhar University, Assiut Branch, Assiut, 71524 Egypt; 2 Division of Biochemistry, Department of Biological Sciences, Faculty of Science, University of Jeddah, Jeddah, Saudi Arabia; 3 Department of Anatomy and Histology, Faculty of Veterinary Medicine, Assiut University, Asyut, Egypt; 4 Department of Pharmacology and Toxicology, Faculty of Pharmacy, Beni-Suef University, Beni-Suef, Egypt; 5 Neuroscience and Geroscience Research Unit, King Fahd Medical Research Center, King Abdulaziz University, Jeddah, Saudi Arabia; 6 Department of Physiology, Faculty of Medicine, Neuroscience Unit, King Abdulaziz University, Jeddah, Saudi Arabia

**Keywords:** Lung cancer, NSCLC, GSK-3β, Wnt/β-catenin, PI3/AKT/mTOR

## Abstract

Lung cancer (LC) primarily affects men and is the third most common cause of cancer-related death worldwide. Treatment for LC involves many phases, each with specific challenges arising from the characteristics of malignancy and the limitations of chemotherapy. GSK-3β, a serine/threonine kinase factor, is essential for several signals that control important cellular functions and a variety of diseases, including cancer. GSK-3β regulates transcription factors through phosphorylation, which in turn activates signaling pathways such as PI3K/AKT/mTOR, Wnt/β-catenin, and NF-κB. These signaling pathways might affect LC. Additionally, targeting GSK-3β offers a promising approach to overcoming drug resistance in cancer therapy. Inhibiting GSK-3β has been demonstrated to boost chemotherapy sensitivity, making cancer cells more responsive to treatment, while also suppressing their growth and survival. We provide an in-depth analysis of scientific literature that investigates the manner in which inhibiting GSK-3β with various compounds might be used to treat, impede, and improve the therapeutic response in LC cells. Next, we suggested that GSK-3β inhibition might be a therapeutic option to increase patient survival in patients with LC. For this reason, further studies on this subject will be required in the coming years.

**Graphical abstract:**

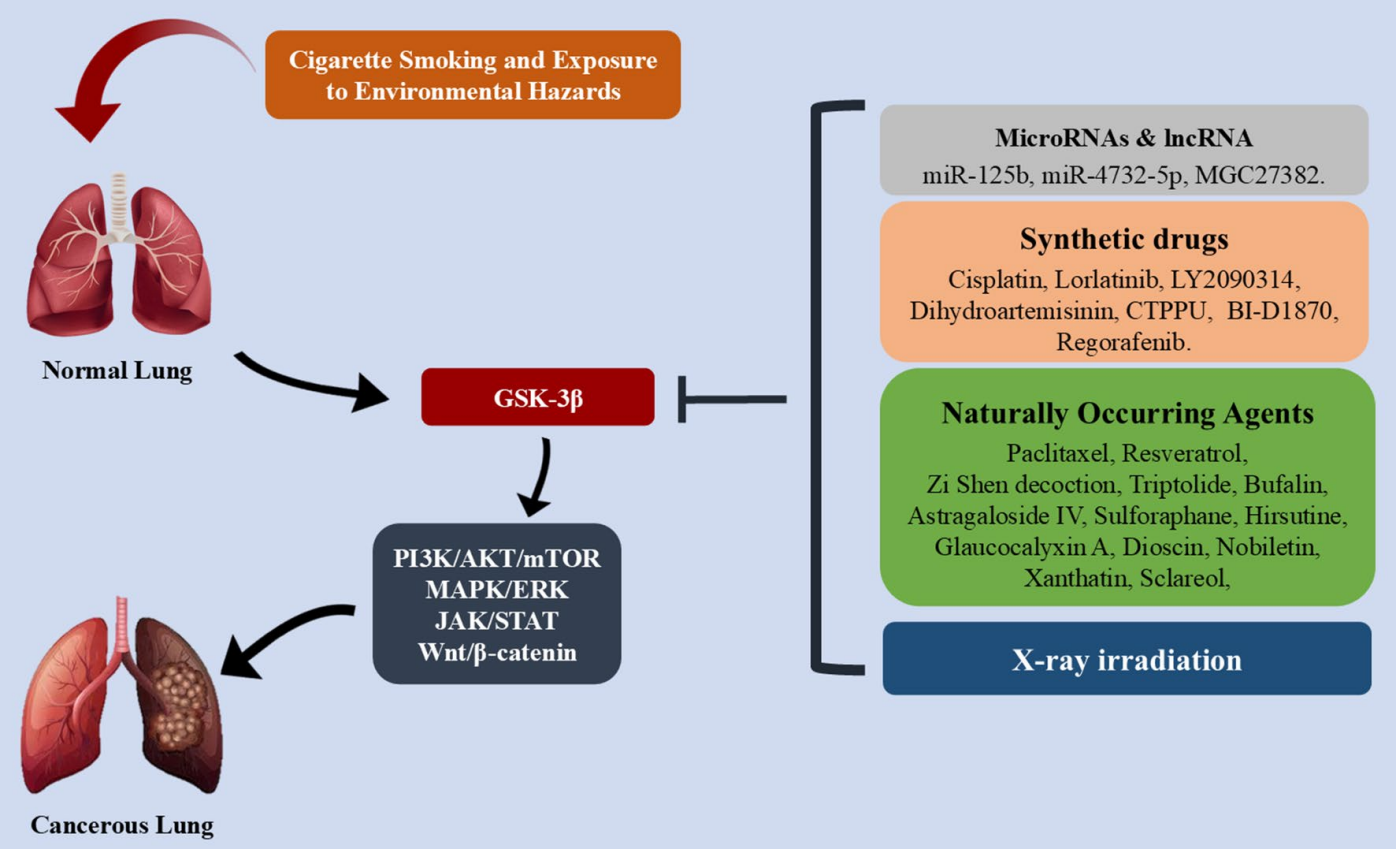

## Introduction

### Lung cancer

Lung cancer (LC) is the leading cause of cancer-related deaths, despite being the third most common cancer after breast and prostate cancer [1–3]. Males are roughly twice as likely as females to die from LC [4]. Since early-stage disease is asymptomatic, more than 70% of cases are identified when the disease has progressed and there is no longer a chance of recovery. Significantly, the incidence and mortality of LC are among the most common cancers, making it a major public health issue [5, 6]. Currently, the 5-year survival rate is low, and this is a major contributing factor to its high mortality rate [7]. Globally, cigarette smoking is the greatest risk factor for LC [8]. In addition, exposure to other environmental and occupational carcinogens, such as radon, asbestos, arsenic, and polycyclic aromatic hydrocarbons, is known to increase the risk of LC [8, 9]. Susceptibility to LC, treatment results, and survival rates may also be influenced by environmental variables, geographic location, and income level [10]. Surgery is the primary treatment option for early-stage LC, and it offers the best chance for prolonged survival [11]. Due to its benefits for survival and lower incidence of side effects, immunotherapy is now the typical of care, either as a monotherapy or in combination with chemotherapy [12].

### ***Classification of lung cancer***

Due to its great heterogeneity, LC can develop in the bronchial tree at multiple sites. It can also be classified as either small-cell lung cancer (SCLC) or non-small-cell lung cancer (NSCLC) based on histological characteristics [13]. Approximately 85% of LC cases are NSCLC, which is further divided into three main histological subtypes: large cell carcinoma, lung squamous cell carcinoma (LUSC) and lung adenocarcinoma (LUAD) [14, 15].

During the last decade, there has been a dramatic improvement in clinical outcomes, mostly owing to the use of targeted therapies in genetically chosen patient subpopulations and the excellent results of immunotherapy in patients who have progressed NSCLC [16]. However, the percentage of NSCLC patients who survive long term is quite low [17]. In contrast to traditional anticancer agents, which affect all rapidly dividing cells, including cancerous and normal cells, molecular targeted therapy uses substances such as hormone therapies, immunotherapies, signaling inhibitors, genetic modulators, apoptosis activators, and angiogenesis inhibitors that treat NSCLC [18, 19]. Several signaling pathways are linked to NSCLC, and it has been demonstrated that some advanced NSCLC tumors are affected by the carcinogenic expression of tyrosine kinases [20, 21].

### Lung carcinogenesis and signaling pathways

The progression of LC is linked to a number of chromosomal abnormalities, such as deletions and amplifications, as well as mutations affecting tumor suppressor genes and oncogenes, which control DNA repair genes and cell proliferation [22]. LC has been linked to a number of targetable genetic changes, including amplifications, structural changes, and mutations in several proto-oncogenes [23].

Transmembrane tyrosine kinase receptors called the epidermal growth factor receptor (EGFR) family promote cell division and improve resistance to chemotherapy and radiotherapy [24]. EGFR recognizes a variety of ligands, such as amphiregulin, transforming growth factor (TGF), and epidermal growth factor (EGF), which, when bound, cause tyrosine residues inside the intracellular domain to undergo conformational changes, dimerization, and autophosphorylation. This creates binding sites for transmission proteins that have phosphotyrosine-binding domains or Src-homology 2 domains. This pathway regulates key cellular processes, including cell proliferation, invasion, metastatic spread, apoptosis, and tumor angiogenesis via downstream targets such as the PI3K/AKT/mTOR, MAPK, and JAK/STAT signaling pathways [25, 26].

The PI3K/AKT/mTOR pathway is an important regulator of cancer growth, metastasis, and therapy failure that connects lipid kinase and tyrosine kinase mechanisms [27, 28]. The initiation of signaling occurs when a ligand binds to phosphorylated residues of tyrosine receptor kinases (RTKs), such as those found in the EGFR family, insulin, and insulin-like growth factor 1 (IGF-1) receptor [29]. Mutations in the PIK3CA gene are a common mechanism of PI3K activation in various cancers, including LC, leading to uncontrolled cell growth and tumor development [30]. Researchers have thoroughly examined the PI3K pathway as possible targets for treating human cancers, which has led to promising molecular therapeutic targets in human tumors [31, 32]. Furthermore, phosphatidylinositol (3,4,5)-trisphosphate (PIP3) is activated by an activated kinase, which attaches to the plasma membrane and catalyzes this process. Next, PIP3 phosphorylates AKT directly or indirectly through the adhesion of phosphoinositide-dependent protein kinase (PDK1), which in turn activates AKT [33, 34]. Additionally, mTORC2 is known to modulate AKT activity in human cells, which, when combined with PDK1-mediated stimulation ring phosphorylation, leads to the full activation of AKT. Through a variety of pathways, active AKT promotes cell growth and survival [35–37]. As a result, in the context of LC, a number of putative inhibitors of the PI3K/AKT/mTOR signal have been developed [38].

Furthermore, AKT inhibits pro-apoptotic Bcl-2 protein family members such as Bcl-2-like protein 4 (BAX) and the NF-κB transcription factor, resulting in enhanced cell survival and antiapoptotic signaling [39, 40]. Bcl-2 is overexpressed in LC to protect cells from apoptosis. Bcl-2 inhibitors exhibit anti-LC efficacy in animal xenograft models [41].

Additionally, the receptor tyrosine kinase family member anaplastic lymphoma kinase (ALK), which controls cell proliferation and is triggered by binding to extracellular ligands, is well known for being the most abundant in NSCLC. ALK consists of a smaller tyrosine kinase domain that carries out mitogenic signal transmission via MAPK and other signaling pathways [42]. The resulting oncoprotein stimulates the ALK signaling pathway, facilitating the survival and multiplication of cells as well as their ability to elude programmed cell death. ALK has been proven to be an effective therapeutic target for a variety of tumor types [43, 44]. All of the abovementioned signals interact with and modulate GSK-3β. We will examine how GSK-3β inhibitors block these signals and their impact on LC modulation.

### Overview of GSK-3β upstream and downstream signaling

Glycogen synthase kinase 3 (GSK-3) is a serine/threonine-directed protein kinase that was discovered in the 1980 s in eukaryotes and was found to influence glycogen synthase activity by phosphorylation, resulting in its inhibition [45, 46]. GSK-3 has two isoforms, GSK-3α and GSK-3β, which are encoded by separate genes [47]. However, in vivo investigations have shown that eliminating GSK-3β can lead to embryo death [48]. GSK3 has been linked to the regulation of many important biological functions, including cell death, differentiation, and proliferation. It has been linked to a variety of illnesses [49–51]. GSK-3 has been shown to play an important role in cancer resistance to chemotherapy, radiotherapy, and targeted treatments [52, 53]. These mechanisms mediated by GSK-3 (by phosphorylation of multiple substrates) indicate that GSK-3 activity must be tightly regulated [46, 54]. Therefore, GSK-3β is considered one of the most valuable targets for treating these disorders [55].

GSK-3, which is ubiquitously expressed, plays an important role in cells by connecting numerous signaling pathways. It is a phosphorylation target for many kinases, and it adjusts its kinase activity to influence various downstream targets [56, 57]. GSK-3 activity is inhibited by phosphorylating a regulatory serine in either isoform, specifically serine 9 in GSK-3β. This is the most well-defined regulatory mechanism [47]. It has also been determined that the phosphorylation of tyrosine residues in GSK-3β changes in a manner dependent on extracellular signals [58].

GSK-3 plays a crucial role in the Wnt/β-catenin and PI3K/Akt signaling pathways, both of which are linked to cancer development [59]. Wnt/β-catenin plays a crucial role in cancer metastasis by promoting proliferation and epithelial-to-mesenchymal transition (EMT). When GSK-3 is active, it can phosphorylate β-catenin at three residues, leading to its proteasomal degradation. This prevents the transcription of important cancer genes, such as CCND1, Myc, and c-jun, which contribute to tumorigenesis and tumor maintenance [60, 61]. Cytoplasmic GSK-3β inhibition in A549 cells may activate the Wnt/β-catenin signal and upregulate survivin expression, possibly leading to resistance to cisplatin (CDDP) in NSCLC cells [62]. Furthermore, during stress, the activated p38MAPK family phosphorylates GSK-3β, thereby reducing its activity. This reduction allows for the regulation of downstream targets, including Wnt signaling and β-catenin, ultimately affecting cellular processes such as survival, proliferation, and differentiation [63–65]. Therefore, genes that affect this pathway play a vital role in maintaining cellular homeostasis.

According to Cui et al. (2013), inhibiting the Wnt pathway with LZTS2 can reduce cell growth and cell cycle progression at the G1/S phase by inhibiting GSK-3β and β-catenin and deactivating the Akt axis [66]. In addition, ZNF185 overexpression reduces the production of vascular endothelial growth factor (VEGF) and matrix metalloproteinase-9 (MMP-9) and blocks the proliferation and invasion of LAC cells by blocking AKT/GSK-3β signaling [67]. He et al. (2017) determined that in A549 and A549/DDP cells, the forced expression of Sox2 inhibited Wnt/β-catenin signaling possibly by upregulating GSK-3β. Sox2 overexpression increased A549 cell clonogenic potential and promoted cell invasion and migration [68].

GSK-3 has been demonstrated to phosphorylate a variety of proteins that are identified as part of the PI3K/AKT/mTOR signaling pathway [69]. AKT affects GSK-3 in various types of cells. GSK-3 phosphorylation is increased when PI3K and AKT are activated in response to different stimuli, but unstimulated GSK-3 phosphorylation is reduced when PI3K and/or AKT are specifically inhibited [70–72]. Momcilovic et al. (2018) demonstrated that lung SCCs utilize the GSK-3β signaling pathway to upregulate glycolysis in response to persistent mTOR inhibition [73]. Additionally, the phosphatase and tensin homolog (PTEN)/Akt/GSK-3β signaling axis controls the expression of invasion markers such as E-cadherin, vimentin, snail, slug, and β-catenin, which in turn control proliferation and EMT [74]. Consequently, inhibiting GSK-3β to target endothelial-to-mesenchymal transition (EndMT) may be a promising therapeutic approach for the treatment of LC [75].

Different factors influence this signal, which increases the level of GSK-3β. In vitro and in vivo, LDHC stimulates the PI3K/AKT/GSK-3β pathway and EMT-related proteins, inducing division, migration, spread, and EMT in LUAD cells [76]. Geng et al. (2017) report MDIG reduces slug, snail, and ZEB1, increasing epithelial markers and adhesion molecules. It also lowers mesenchymal markers, such as vimentin and N-cadherin, which help prevent NSCLC invasion and metastasis. MDIG inhibits GSK-3β phosphorylation and destabilizes β-catenin [77]. GSK-3β is activated via CNPY2 overexpression. This promotes EMT by increasing Snail and decreasing E-cadherin [78]. Similarly, Guo et al. reported that KIF2C overexpression increased the levels of β-catenin, p-GSK-3β, and phosphorylated p-AKT, which in turn blocked NSCLC cell death and increased the invasion, migration, and proliferation of NSCLC cells [79]. However, in 2022, Chen et al. showed that KIF26B affects the AKT/GSK3-β/β-catenin pathway, which in turn has a tumor-suppressive effect on NSCLC [80].

GSK3-β is necessary for NF-κB activity and can control NF-κB activity by phosphorylating NF-κB essential modifier (NEMO) with IκB kinases (IKK) [81]. Moreover, β-catenin and GSK-3β protein expression may be regulated by the STAT3 signaling system [82]. Furthermore, SLIT2 and roundabout guidance receptor 1** (**ROBO1) have opposite effects on SCLC tumors. SLIT2 slows growth, while ROBO1 promotes it, by regulating the TGF-β1/GSK3-β/β-catenin signaling pathway in tumor cells and tumor-associated macrophages (TAMs) [83].

**Fig. 1:**
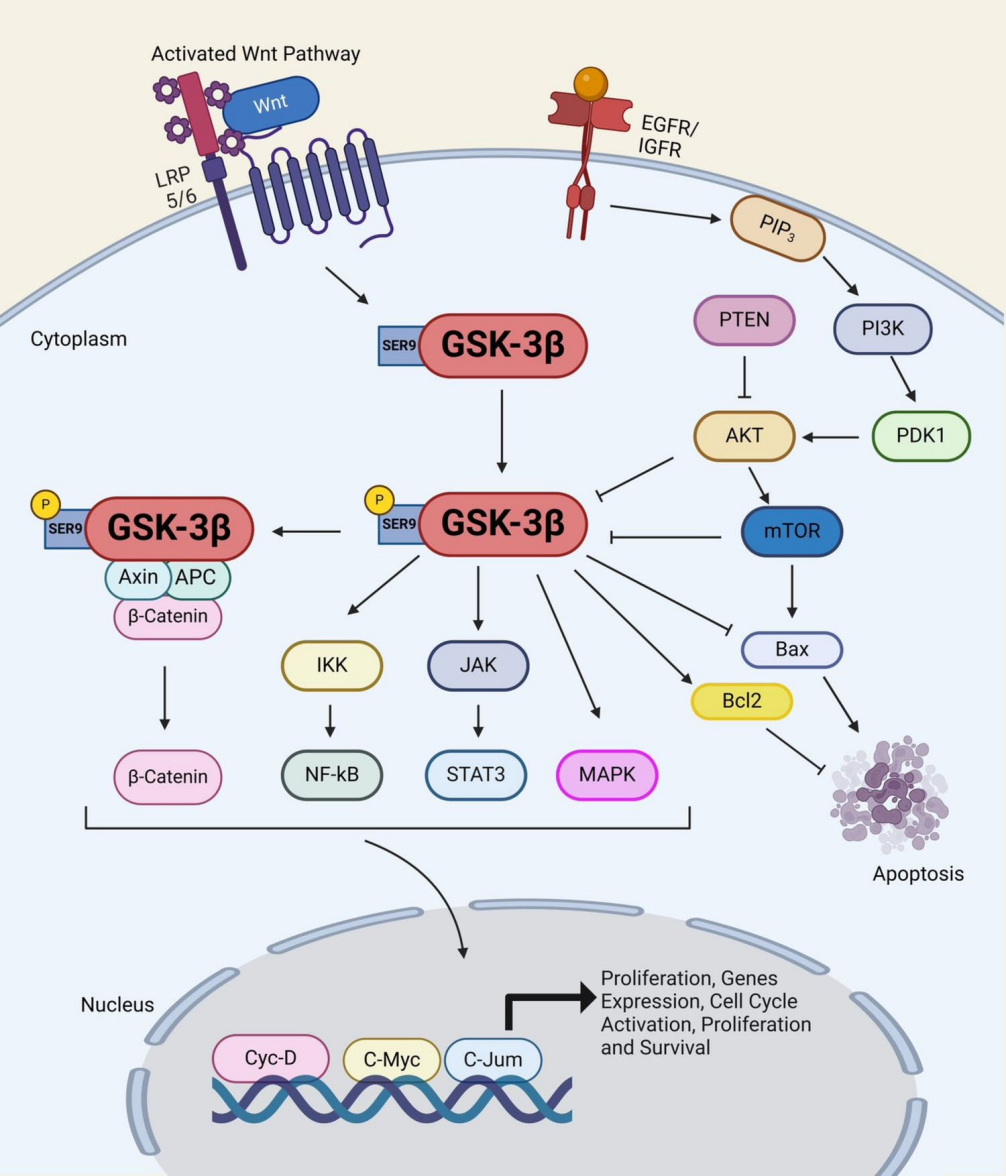
GSK-3β by signaling pathways. The pathway regulates cellular processes, including proliferation, invasion, metastasis, apoptosis, and angiogenesis, through downstream targets like PI3K/AKT/mTOR, MAPK, and JAK/STAT pathways. Transmembrane tyrosine kinase receptors like the epidermal growth factor receptor (EGFR) family promote cell division and resistance to chemotherapy and radiotherapy. GSK-3 activity is inhibited by phosphorylating serine 9 in GSK-3β, a key regulatory mechanism. Phosphorylation of tyrosine residues varies with extracellular signals. GSK-3 influences Wnt/β-catenin and PI3K/AKT pathways, both linked to cancer. Wnt/β-catenin promotes cancer metastasis by encouraging proliferation and EMT. Active GSK-3 phosphorylates β-catenin, leading to its degradation, which suppresses cancer gene transcription (like CCND1, Myc, c-jun), aiding tumor growth and maintenance. Created in BioRender. Alghamdi, B. (2024) https://BioRender.com/g79g502. (Agreement no: AJ27QBH7GE)

Ultimately, dysregulation of many GSK3-mediated signaling pathways and oncogenic activation of tyrosine kinases are linked to the development of LC [84, 85]. GSK-3β knockdown in NSCLC cell lines reportedly causes apoptosis, limits cell motility, arrests tumor cells in the G0/G1 phase, and suppresses cell proliferation [86]. As a result, controlling GSK-3β by signaling pathways affects both its expression and cellular homeostasis. (Fig. 1).

In this review, we provide an in-depth analysis of scientific literature that investigates the manner in which inhibiting GSK-3β with various agents might be used to treat, impede, and improve the therapeutic response in LC cells.

## Targeting GSK-3β to treat different LCs

**Fig. 2:**
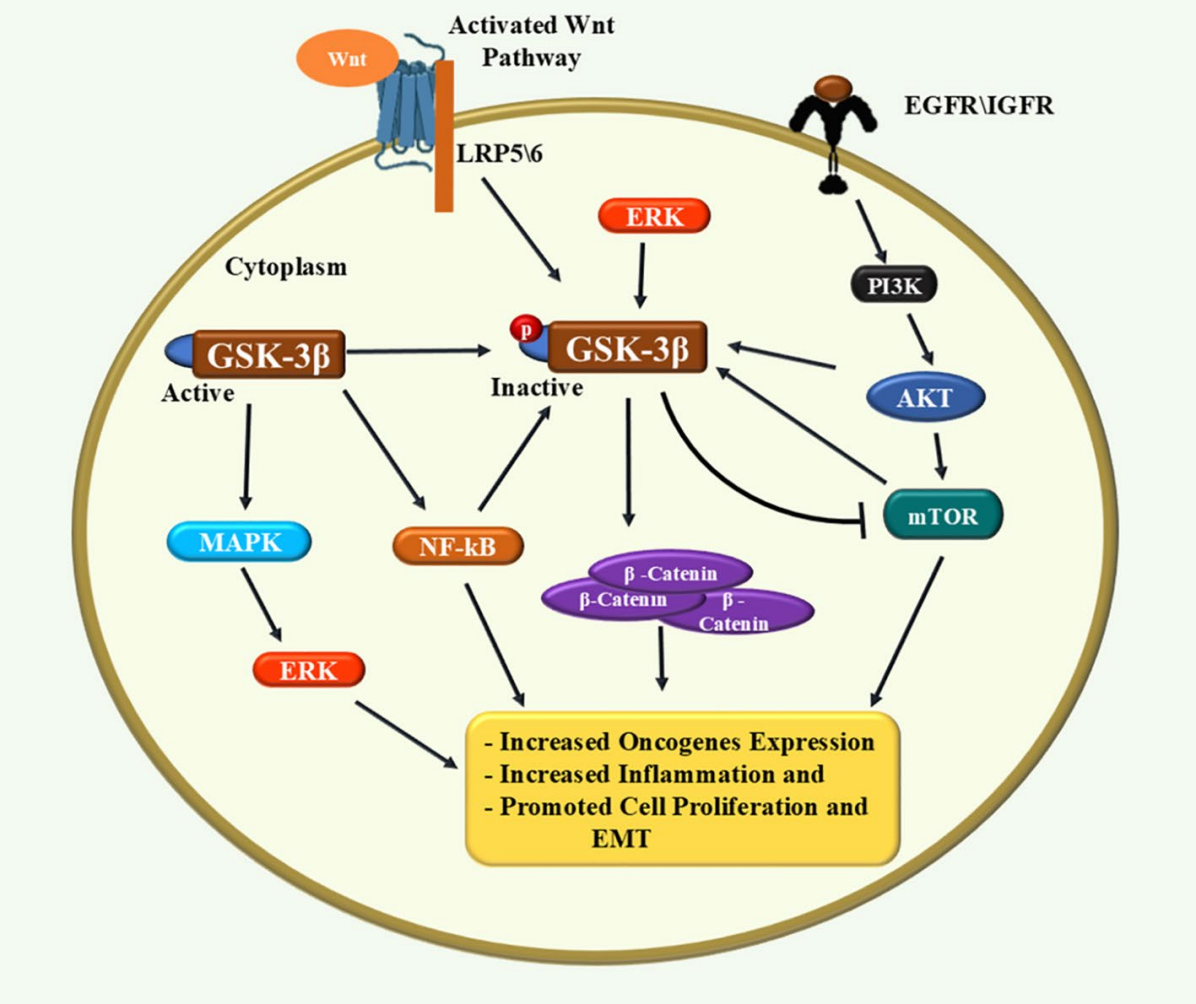
The proposed mechanism for the role of GSK-3β in lung cancer. GSK-3β’s role in lung cancer involves multiple pathways. It collaborates with PI3K/AKT, where AKT inhibits GSK-3β, increasing β-catenin and promoting cell survival. GSK-3β phosphorylates β-catenin, leading to degradation; when inhibited, β-catenin accumulates and activates Wnt genes. It also influences mTOR, regulating growth, and affects NF-κB, which evokes inflammation and is regulated by NF-κB. GSK-3β interacts with MAPK/ERK, influencing proliferation, with ERK modulating GSK-3β activity

Briefly, GSK-3β participates in various signals, playing a central role in cancer development and progression [87]. It cooperates with the PI3K/AKT pathway, where AKT phosphorylates and inhibits GSK-3β, leading to increased β-catenin and oncogenic protein activity, thereby encouraging cell survival and growth. GSK-3β also phosphorylates β-catenin in the Wnt/β-catenin signal, marking it for degradation. When GSK-3β is inhibited, β-catenin accumulates and activates Wnt target genes [87, 88]. Additionally, GSK-3β influences mTOR activity, affecting cell growth and proliferation, while mTOR also modulates GSK-3β, forming a feedback loop [89]. Moreover, GSK-3β impacts NF-κB activity, which evokes inflammation, and it is itself regulated by NF-κB. Finally, GSK-3β interacts with the MAPK/ERK pathway, affecting cell proliferation and survival, with ERK phosphorylating and controlling GSK-3β activity [48, 90]. The proposed mechanism for the role of GSK-3β in lung cancer was illustrated in Fig. 2.

Additionally, targeting GSK-3β offers a promising approach to overcoming drug resistance in cancer therapy. Inhibiting GSK-3β has been demonstrated to boost chemotherapy sensitivity, making cancer cells more responsive to treatment, while also suppressing their growth and survival. This reduces the risks of drug resistance and metastasis [52, 53, 91–93].

### RNAs modulate GSK-3β

#### MicroRNAs (miRNAs)

MicroRNAs (miRNAs), which are small noncoding single-stranded RNAs, play a crucial role in gene control after transcription [94]. Accumulating evidence has demonstrated that miR-410-3p plays a vital role in cancer progression [95]. Wang et al. reported that downregulating miR-125b using the PI3K inhibitor LY294002 inhibits cell proliferation, suppresses p-GSK-3β, Wnt, and β-catenin protein production, and promotes caspase-3 activity in A549 cells. Therefore, it promotes apoptosis and inhibits cell growth and proliferation [96]. Similarly, overexpression of miR-4732-5p suppresses LUAD migration, invasion, and metastasis in vitro and in vivo by inhibiting the PI3K/AKT/GSK-3β/Snail pathway produced by EGF-driven EMT and xenotropic and polytropic retrovirus receptor 1 (XPR1) knockdown [97]. Furthermore, both in vitro and in vivo, EGF-like growth factor Amphiregulin (AREG) preserved regulatory T cells (Treg) suppressive function through the EGFR/GSK-3β/Forkhead Box P3 (Foxp3) axis. Moreover, GSK-3β activity was restored, and Treg cell function was decreased by EGFR suppression with the tyrosine kinase antagonist gefitinib [98].

#### Long noncoding RNA (lncRNA)

lncRNA is a type of nucleotide sequence that has the capacity to code for no more than 200 nucleotides. It has been shown to participate in both tumor-suppressive and oncogenic pathways to perform a variety of biological activities in cancer [99, 100]. Furthermore, according to Li et al., MGC27382 is an lncRNA whose expression is downregulated in NSCLC, and it inhibits the growth of NSCLC by blocking the AKT/GSK-3β pathway and suppressing AKT and GSK-3β phosphorylation. Thus, overexpressing MGC27382 could be an effective strategy for preventing the progression of NSCLC [101]. Moreover, miR-3909 and miR-3681-3p were competitively sponged by circ-GSK-3β to increase circ-GSK-3β expression. Circ-GSK-3β may inhibit the ability of circ-GSK-3β to inhibit the binding of FKBP51 to GSK-3β to prevent the phosphorylation of GSK-3βS9, which would inactivate Wnt/β-catenin signaling. This study concluded that circ-GSK3B suppressed LUAD development through up-regulating and activating GSK-3β [102].

### Drugs modulate cellular signaling to inhibit GSK-3β

#### Synthetic drugs

##### Cisplatin

Cisplatin has been clinically demonstrated to effectively treat several cancer types, such as sarcomas, as well as tumors of the blood vessels, soft tissues, bones, and muscles [103, 104]. Because DVL2 is a major controller of the Wnt/β-catenin signal, downregulating the Wnt/β-catenin signaling pathway through the inhibition of DVL2 or its inhibitor shDVL2 resensitized A549/CDDP cells to CDDP. Furthermore, p-GSK-3β was aberrantly downregulated by DVL2 [105].

##### Lorlatnib

Lorlatinib is an orally available, ATP-competitive molecule inhibitor of tyrosine kinase receptors that was recently approved for treating NSCLC [106]. According to study conducted by Shimizu et al., GSK-3β is essential for the development of acquired resistance to lorlatinib in ALK-positive NSCLC, which is mediated by lorlatinib intermediate-resistant cells. Furthermore, cells with acquired resistance generated from patients treated with alectinib, regardless of whether they had secondary mutations, were also sensitized to lorlatinib via GSK-3β inhibition. GSK-3β inhibitors thereby prevent NSCLC from becoming resistant to lorlatinib [92]. These findings suggest that GSK-3β may be a useful molecular target for preventing the development of acquired lorlatinib resistance and overcoming ALK-TKI resistance.

##### LY2090314

LY2090314 has been demonstrated to be an effective, selective GSK-3 inhibitor, and preliminary safety investigations have been performed in a first-in-human, phase I dose progression study evaluating its therapeutic efficacy in patients suffering from advanced solid tumors [107]. The third-generation EGFR-TKI osimertinib is taken orally and is commonly authorized for use as a first-line treatment for advanced NSCLC with activating EGFR mutations [108]. Preclinical studies using lung cancer cells resistant to osimertinib revealed that EMT was linked to both elevated ZEB1 expression and decreased microRNA-200c. To combat EMT-associated resistance to osimertinib in EGFR-mutant lung cancer, the use of GSK-3 inhibitors, such as LY2090314, significantly reduced the proliferation and promoted the death of resistant cells [93].

##### Dihydroartemisinin

Dihydroartemisinin (DHA), a semisynthetic analog of artemisinin, has also been largely regarded as the best treatment for malaria [109]. According to previous studies, DHA also has anti-inflammatory and anticancer properties [110, 111]. Furthermore, DHA has been shown to increase apoptosis while decreasing proliferation and migration in a variety of cancer types [112, 113]. DHA decreased the ability of the AKT/GSK3-β/cyclin D1 signaling pathway to proliferate in the A549 lung cancer cell line. DHA-treated A549 cells experienced G1 phase cell cycle arrest, which was associated with apparent downregulation of PCNA and cyclin D1 mRNA and protein levels [114].

##### BI-D1870

BI-D1870 (BID) is an ATP-competitive ribosomal S6 kinase (RSK) modulator that selectively inhibits various subtypes of RSK proteins [115]. Abdulrahman and colleagues (2016) reported that BID treatment of A549 cells resulted in a decrease in Bcl2 mRNA expression. Additionally, OPN and phospho-GSK-3β protein expression were reduced by BID [116].

##### *N,N’*-Diarylurea derivatives (CTPPU)

CTPPU has drawn much attention because of its expanding use in medicinal chemistry and drug design, as it has anticonvulsant, anti-inflammatory, and anticancer properties [117, 118]. Through the AKT/GSK-3β/c-Myc signaling pathway, CTPPU induces G1/S cell cycle arrest, which significantly reduces the growth of NSCLC cells, including H460, A549, and H292. Treatment with CTPPU decreased the levels of AKT and its downstream effectors, which include phosphorylated GSK-3β (Ser9), β-catenin, and c-Myc [119].

##### Regorafenib

Regorafenib is a small-molecule inhibitor that targets various membrane-bound and intracellular kinases involved in processes such as tumor angiogenesis, oncogenesis, and maintaining the tumor microenvironment, affecting both pathological and normal cellular functions [120]. Regorafenib has demonstrated anticancer, anti-angiogenic, anti-proliferative, and antimetastatic properties against a range of cancer types [121–123]. Regorafenib could be a potential treatment for LSCC due to its inhibition of GSK-3β using NCI-H1703, NCI-H2170 and SK-MES-1 cell lines [124].

#### Naturally occurring agents

##### Paclitaxel

Paclitaxel is a naturally occurring tricyclic diterpenoid molecule found in the bark and needles of *Taxus brevifolia* [125, 126]. Paclitaxel promotes tubulin assembly into microtubules and inhibits microtubule dissociation, which blocks cell cycle progression, prevents mitosis, and inhibits the growth of cancer cells, in contrast to other tubulin-binding anticancer drugs that control this process [127]. Additionally, paclitaxel and the GSK-3 inhibitor CHIR99021 work together to suppress the growth of H1975 and H1299 NSCLC cell lines both in vitro and in vivo. This may be due to convergent mechanisms of action on microtubule spindle stability, which in turn affects chromosomal alignment during metaphase [128].

##### Resveratrol

Resveratrol is a phytochemical present in numerous foods, including grapes, peanuts, blueberries, and red wine. Resveratrol has numerous bioactivities, including immunomodulatory, anti-inflammatory, and antioxidant properties [129, 130]. Resveratrol has been shown to act as a cancer prevention drug during the primary stages of carcinogenesis [131]. Similarly, a study using the lung cancer cell lines A549 and ASCT-a-1 revealed that resveratrol treatment led to a significant decrease in AKT phosphorylation, which in turn caused a suppression in GSK-3β phosphorylation and an increase in lung cancer cell death [132].

##### Zi Shen decoction

In lung cancer cells, Zi Shen decoction (ZSD), a traditional Chinese medicine, exhibited anticancer effects by regulating the AKT/GSK3-β/β-catenin pathway. ZSD, administered orally, inhibited the growth of Lewis LC in a subcutaneous allograft model, increased necrosis and inflammatory cell proliferation in the tumor tissues, and caused LC cells to undergo apoptosis by downregulating the levels of p-AKT, p-GSK-3β, β-catenin, and Bcl-2. These effects were caused by decreased PI3K and AKT enzyme expression in ZSD, both in vivo and in vitro [133].

##### Triptolide

Triptolide, a significant diterpene active component in T. wilfordii, has been shown to have considerable immunosuppressive and anticancer effects [134, 135]. Triptolide has been shown to suppress the GSK-3β phosphorylation, which in turn slows tumor growth and reverses EMT in Taxol-resistant LUAC. In Taxol-resistant A549 cells, triptolide induced apoptosis, S-phase cell cycle arrest, and inhibited cell growth. Furthermore, the intraperitoneal injection of triptolide into mice significantly delayed tumor growth without clearly causing systemic damage [136].

##### Xanthatin

Xanthatin is a lactone extracted from Xanthium strumarium L. It has been demonstrated to have anti-proliferative, anti-angiogenic, and pro-apoptotic effects in a range of cancers [137]. Tao et al. demonstrated that the anticancer effects of xanthatin on NSCLC require the interruption of GSK-3β activity in conjunction with the preferential inhibition of constitutive STAT3 activation [138].

##### Hirsutine

Hirsutine, a significant indole alkaloid obtained from U. rhynchophylla, has garnered considerable attention because of its diverse bioactivities [139]. In the A549 xenograft mouse model, hirsutine-mediated GSK-3β dephosphorylation and mitochondrial apoptosis were significantly influenced by disruption of the ROCK1/PTEN/PI3K/AKT signal. Hirsutine predominantly induces apoptosis by activating PTEN and ROCK1, inactivating PI3K/AKT, which then triggers GSK-3β dephosphorylation and the opening of the mitochondrial permeability transition pore, which in turn triggers caspase-3 activation and death [140].

##### Pristimerin

Pristimerin is a quinonemethide triterpenoid that has been isolated from many species belonging to the Hippocrateaceae and Celastraceae families [141]. Pristimerin has been shown to have numerous pharmacological benefits, including anti-inflammatory, antitumor, antioxidant, and antibacterial effects [142, 143]. In A549 and NCI H446 cells, pristimerin caused G0/G1 arrest, cell death, and inhibition of cell growth. Pristimerin and CDDP work in concert to cause cell death by inhibiting autophagy and downregulating p-GSK3β and p-AKT, thereby blocking the microRNA 23a/AKT/GSK-3β signaling pathway [144].

##### Astragaloside IV (AS-IV)

AS-IV is an astragalus polysaccharide with exceptional potency [145]. AST IV may exhibit anti-inflammatory, antifibrotic, and anticancer properties [146–148]. By suppressing the AKT, GSK-3β, and β-catenin signaling axis, AS-IV significantly increased cell death in NSCLC cells. Moreover, there was a downregulation in Bcl-2 and an upregulation of Bax, a hallmark of cell death. Additionally, AS-IV stimulates the cleavage of caspase-3, another indication of apoptosis [149].

##### Sulforaphane

One of the most investigated bioactive, anti-inflammatory compounds is organosulfur isothiocyanate sulforaphane, which is present in Brassicaceae plants, particularly broccoli. It has been proposed that sulforaphane can cause the production of detoxification enzymes, cell cycle arrest, apoptosis, and epigenetic control [150, 151]. While sulforaphane reduced the levels of miR-616-5p through histone modifications, it also inactivated the GSK-3β/β-catenin signal [152].

##### Glaucocalyxin A

Cymbidium calyx is the source of the diterpenoid chemical known as glaucocalyxin A (GLA) [153]. It has also been shown that GLA possesses immune-suppressive, anti-inflammatory, and antitumor properties [154, 155]. According to Zhang et al., the anticancer effect of GLA in NSCLC is mediated by suppressing the phosphorylation of PI3K, AKT, and GSK-3β, which leads to apoptosis. These findings suggest that GLA may be a potential natural treatment for NSCLC. GLA caused apoptosis and reduced the survival rate of cancer cells. GLA enhanced caspase 3 cleavage and downregulated Bcl-2 expression while upregulating Bax expression. Furthermore, a two-day infusion of GLA markedly reduced the development of A549 xenograft tumors while concurrently increasing apoptosis and decreasing proliferation [156].

##### Bufalin

Bufalin, the primary active component of traditional Chinese medicine Chansu, is a steroid derivative extracted from the epidermis and parotid venom glands of toads [157]. Research has demonstrated that bufalin has antitumor efficacy against several malignancies, such as LC [158, 159]. According to Kang et al., bufalin may induce apoptosis in H1975 cells by enhancing proteasomal degradation of Mcl-1 through downregulation of p-GSK-3β [160].

##### Sclareol

Sclareol from Salvia sclarea L (SSL, S. sclarea), often known as clary sage, is important in herbal medicine and essential oil operations [161]. Notably, a variety of bioactivities, such as antioxidant, antibacterial, antifungal, anti-inflammatory, and antidiabetic properties, have been studied [162]. Furthermore, Pan et al., 2020 showed that the administration of CDDP in conjunction with sclareol markedly reduced the ability of A549 cells to migrate and survive. In this study, the total and phosphorylation levels of key signaling molecules, including AKT, GSK-3β, ERK1/2, and JNK, which regulate AP1 and Snail, were examined. After 24 h of sclareol treatment, A549 cells showed lower total levels of AKT and JNK proteins, while ERK1/2 and GSK3β levels stayed the same. However, short-term exposure to sclareol (20 min) reduced phosphorylation levels of most tested molecules, except ERK1/2. Additionally, an in vivo investigation revealed that the combination of sclareol and cisplatin had the greatest antitumor efficacy [163]. Consequently, sclareol can be used as an adjuvant drug to boost the effectiveness of cisplatin in NSCLC chemotherapy.

##### Dioscin

Dioscin is a steroidal saponin with exceptional anti-inflammatory, antioxidative, lipid-lowering, and antitumor properties [164, 165]. Interestingly, through suppression of AKT, mTOR, and GSK-3β signaling phosphorylation, dioscin inhibited the proliferation, invasion, migration, and EMT of lung adenocarcinoma cells. This was likely achieved by binding to AKT and mTOR and phosphorylating them without changing the overall amount of protein in the cells. These alterations are enhanced by the PI3K inhibitor LY294002 [166].

##### Nobiletin

Nobiletin is a prominent component of polymethoxylated flavones in citrus fruit peels [167]. The advantageous health characteristics of nobiletin, such as its anticarcinogenic effects on several cancer types, have recently garnered much attention [168]. A study found that nobiletin increases chemosensitivity to adriamycin by modulating the AKT/GSK-3β/β-catenin pathway in A549 human NSCLC cells, effectively reducing phosphorylation of β-catenin and GSK3β [91].

### X-ray irradiation

Currently, radiation is one of the main strategies used in the treatment of LC [169]. Both in vivo and in vitro, X-ray irradiation increased autophagy and decreased GSK-3β expression. Consequently, GSK-3β suppresses autophagy and enhances the radiosensitivity of NSCLC cells. Moreover, NSCLC differentiation was positively correlated with GSK-3β expression, and in 89 NSCLC patients, negative GSK-3β expression was linked to a better prognosis. Following X-ray irradiation, NSCLC tissues exhibit decreased expression levels of GSK-3β and p62 and increased expression of the autophagy-related protein LC3 [170]. On the other hand, investigations by Liu et al. showed that radiation-induced EMT is regulated by TBK1 (a noncanonical IKK family member that exhibits homology to the kinase IkB), which governs GSK-3β activation and ZEB1 expression [171]. Therefore, TBK1 could be a helpful target for the treatment of diseases resulting from radiotherapy-induced metastases.

Table 1 summarizes the effects of different agents that inhibited GSK-3β signaling in lung cancer.

**Table 1:** Table for summarizing the effects of different agents that inhibited GSK-3β signaling in lung cancer

Agent	Class	Model	Main effects	Reference
Cisplatin	Synthetic	A549	Since DVL2 plays a key role in regulating the Wnt/β-catenin signaling pathway, its inhibition or the suppression of its inhibitor, shDVL2, restored sensitivity to CDDP in A549/CDDP cells. Additionally, DVL2 caused downregulation in p-GSK-3β	[105]
Lorlatnib	Synthetic	Intermediate resistant cells from a patient-derived cell model	Cells with acquired resistance generated from patients treated with alectinib, regardless of whether they had secondary mutations, were also sensitized to lorlatinib via GSK-3β inhibition GSK-3β inhibitors thereby prevent NSCLC from becoming resistant to lorlatinib	[92]
LY2090314	Synthetic	Osimertinib-resistant lung cancer cells	Lung cancer cells resistant to osimertinib revealed that EMT was linked to both elevated ZEB1 expression and decreased microRNA-200c GSK-3 inhibitors, such as LY2090314, significantly reduced the proliferation and promoted the death of resistant cells	[93]
Dihydroartemisinin	Semisynthetic analog of artemisinin	A549 lung cancer cell line	DHA decreased the ability of the AKT/GSK3-β/cyclin D1 signaling pathway to proliferate in the A549 lung cancer cell line. DHA-treated A549 cells experienced G1 phase cell cycle arrest, which was associated with apparent downregulation of PCNA and cyclin D1 mRNA and protein levels	[114]
BI-D1870	Synthetic	A549 lung cancer cell line	BID treatment of A549 cells resulted in a decrease in Bcl2 mRNA expression. Additionally, OPN and phospho-GSK-3β protein expression were reduced by BID	[116]
*N,N’*-Diarylurea Derivatives (CTPPU)	Synthetic	H460, A549, and H292 cell lines	CTPPU induces G1/S cell cycle arrest, which significantly reduces the growth of NSCLC cells Treatment with CTPPU decreased the levels of AKT and its downstream effectors, which include phosphorylated GSK-3β, β-catenin, and c-Myc	[119]
Regorafenib	Synthetic	NCI-H1703, NCI-H2170 and SK-MES-1 cell lines	Regorafenib could be a potential treatment for LSCC due to its inhibition of GSK-3β	[124]
Paclitaxel	Natural	H1975 and H1299 NSCLC cell lines both in vitro and in vivo	Paclitaxel and the GSK-3 inhibitor CHIR99021 work together to suppress the growth of H1975 and H1299 NSCLC cell lines both in vitro and in vivo	[128]
Resveratrol	Natural	A549 and ASCT-a-1 cell lines	Resveratrol treatment led to a significant decrease in AKT phosphorylation, which in turn caused a suppression in GSK-3β phosphorylation and an increase in lung cancer cell death	[132]
Zi Shen decoction (ZSD),	Natural	Lewis LC in a subcutaneous allograft model	In lung cancer cells, Zi Shen decoction (ZSD), a traditional Chinese medicine, exhibited anticancer effects by regulating the AKT/GSK3-β/β-catenin pathway. ZSD, administered orally, inhibited the growth of Lewis LC in a subcutaneous allograft model, increased necrosis and inflammatory cell proliferation in the tumor tissues, and caused LC cells to undergo apoptosis by downregulating the levels of p-AKT, p-GSK-3β, β-catenin, and Bcl-2	[133]
Triptolide	Natural	Taxol-resistant A549 cells and in vivo mice model	Triptolide has been shown to suppress the GSK-3β phosphorylation, which in turn slows tumor growth and reverses EMT in Taxol-resistant LUAC In Taxol-resistant A549 cells, triptolide induced apoptosis, S-phase cell cycle arrest, and inhibited cell growth Intraperitoneal injection of triptolide into mice significantly delayed tumor growth without clearly causing systemic damage	[136]
Xanthatin	Natural	A549, H1975, H1650, and HCC827 cell lines	Anticancer effects of xanthatin on NSCLC require the interruption of GSK-3β activity in conjunction with the preferential inhibition of constitutive STAT3 activation	[138]
hirsutine	Natural	A549 xenograft mouse model	In the A549 xenograft mouse model, hirsutine-mediated GSK-3β dephosphorylation and mitochondrial apoptosis were significantly influenced by disruption of the ROCK1/PTEN/PI3K/AKT signal. Hirsutine predominantly induces apoptosis by activating PTEN and ROCK1, inactivating PI3K/AKT, which then triggers GSK-3β dephosphorylation and the opening of the mitochondrial permeability transition pore, which in turn triggers caspase-3 activation and death	[140]
Pristimerin	Natural	A549 and NCI H446 cell lines	In A549 and NCI H446 cells, pristimerin caused G0/G1 arrest, cell death, and inhibition of cell growth Pristimerin and CDDP work in concert to cause cell death by inhibiting autophagy and downregulating p-GSK3β and p-AKT, thereby blocking the microRNA 23a/AKT/GSK-3β signaling pathway	[144]
Astragaloside IV	Natural	HCC827, A549, and NCI-H1299	By inhibiting the AKT, GSK-3β, and β-catenin signaling pathways, AS-IV markedly enhanced cell death in NSCLC cells AS-IV decreased Bcl-2 levels while increasing Bax, a key marker of apoptosis AS-IV promotes caspase-3 cleavage, further confirming apoptosis induction	[149]
Sulforaphane	Natural	H1299, 95 C and 95D	While sulforaphane reduced the levels of miR-616-5p through histone modifications, it also inactivated the GSK-3β/β-catenin signal	[152]
Glaucocalyxin A	Natural	A549 xenograft tumors	Anticancer effect of GLA in NSCLC is mediated by suppressing the phosphorylation of PI3K, AKT, and GSK-3β, which leads to apoptosis GLA caused apoptosis and reduced the survival rate of cancer cells. GLA enhanced caspase 3 cleavage and downregulated Bcl-2 expression while upregulating Bax expression A two-day infusion of GLA markedly reduced the development of A549 xenograft tumors while concurrently increasing apoptosis and decreasing proliferation	[156]
Bufalin	Natural	H1975 cell lines	Bufalin induce apoptosis by enhancing proteasomal degradation of Mcl-1 through downregulation of p-GSK-3β	[160]
Sclareol	Natural	A549 cell lines and xenograft tumor model	Administration of CDDP in conjunction with sclareol markedly reduced the ability of A549 cells to migrate and survive After 24 h of sclareol treatment, A549 cells showed lower total levels of AKT and JNK proteins, while ERK1/2 and GSK3β levels stayed the same. However, short-term exposure to sclareol (20 min) reduced phosphorylation levels of most tested molecules, except ERK1/2. Additionally, an in vivo investigation revealed that the combination of sclareol and CDDP had the greatest antitumor efficacy	[163]

## Conclusions and future recommendations

LC is one of the most common malignancies, with a high death rate globally. Because there are many risk factors for cancer, LC is associated with several risk factors that trigger intracellular signals and stimulate transcription factors that lead to cell division and the initiation of carcinogenesis. Among these regulatory molecules is GSK-3β, which modulates many signaling cascades to govern cell viability and activity. As we previously discussed, studies have shown that GSK-3β inhibition prevents the onset of carcinogenesis, while its activation is associated with its progression. According to the reviewed studies, GSK-3β inhibition may be a potential target for LC therapy; as such, this topic merits careful investigation, especially in the context of ineffective and unsafe existing LC treatments. Furthermore, targeting GSK-3β presents a promising strategy to overcome drug resistance in cancer treatment. Inhibiting GSK-3β has been shown to enhance the sensitivity of cancer cells to chemotherapy, making them more responsive to therapy, and to suppress their growth survival. In conclusion, this review broadens our therapeutic options beyond a limited set of treatments for LC. Given this, significant efforts should be put into developing these promising ingredients in various dosage forms for use in both animals and humans, with the goal of translating lab findings into real-world applications and continuing to see surprising results.

## Data Availability

Data sharing not applicable to this article as no datasets were generated or analyzed during the current study.
